# Supplementary material for: Patterns of Intron Gain and Loss in Fungi
Source: PLoS Biol. 2004 Nov 30;2(12):e422. doi: 10.1371/journal.pbio.0020422 (PMC532390; doi:10.1371/journal.pbio.0020422)
Supplement: Table S1 — Also available at http://genes.mit.edu/NielsenEtAl/. (4.3 MB ZIP). [file pbio.0020422.st001.zip › NielsenEtAl/html/783.html]

AN2315.1.NCU05430.1.MG03185.1.FG04312.1


```
 CLUSTAL W (1.82) Multiple Sequence Alignments - Introns Inserted


Sequence 1: NCU05430.1	520 aa
Sequence 2: FG04312.1	513 aa
Sequence 3: MG03185.1	522 aa
Sequence 4: AN2315.1	513 aa
Alignment Length: 528 aa
Number Identitical Residues: 423 aa
Alignment Score (without introns) 16148


MG03185.1 	-MFKS2GLSPIARAARP----ASRLAARPSLRLPKYSLGARFASTQGVGDGKIHQ0VIG~
NCU05430.1	MSQYS~GISAFARTARPSFAAASRRAVRPAALNLRAPALSRFASSAGVGDGKIYQ~VIG1
FG04312.1 	-MFKS2GISSFARAARP--AVLPRRALRPSAL--RLPISSRWAS-TGVGAGKIHQ0VIG~
AN2315.1  	MARTF~GRAAFAR-PSP----VARRALQPSKLN-GFPSFARLASTETAAGGKIHQ~VIG~
          	      * :.:** . *     .* * :*:      .  :* **:  .. ***:* *** 

MG03185.1 	AVVDGEHYPIINL1KFDTAKLPPILNALETTNNNQKLVLEVS0QHLGENVVRCIAMDG1T
NCU05430.1	AVVD--------V1KFDTDKLPPILNALETQNNGQKLVLEVS0QHLGENVVRCIAMDG~T
FG04312.1 	AVVD--------V1KFDGAKLPAILNSLECQNNGQKLVLEVS0QHLGESVVRCIAMDG1T
AN2315.1  	AVVD--------V1KFEGEKLPAILNAIETENNGQKLVLEVS0QHLGENVVRTIAMDG1T
          	****        : **:  ***.***::*  **.******** *****.*** ***** *

MG03185.1 	EGLTRGAKAKDT~GAPITIP~VGSGTLGRIMNVTGDPIDERGPIKTDKYLPIHAEAPEFV
NCU05430.1	EGLVRGAKASDT1GAPITIP2VGPATLGRIINVTGDPIDERGPIKTDKFRPIHAEAPEFV
FG04312.1 	EGLVRGATVQDT~GAPITIP~VGPATLGRIMNVTGDPIDERGPIKTDKRLPIHTEAPEFV
AN2315.1  	EGLTRGAPARDT~GAPITIP~VGPGTLGRIVNVTGDPIDERGPIKATKYAPIHAEAPEFT
          	***.*** . ** ******* **..*****:**************: *  ***:*****.

MG03185.1 	EQSTTAEILVTGIKVVDLLAPYARGGKIGLFGGAGVGKTVFIQELI~NNIAKAHGGYSVF
NCU05430.1	EQSTTAEILVTGIKVVDLLAPYARGGKIGLFGGAGVGKTVFIQELI~NNIAKAHGGYSVF
FG04312.1 	EQSTSAEVLVTGIKVVDLLAPYARGGKIGLFGGAGVGKTVFIQELI~NNIAKAHGGYSVF
AN2315.1  	EQSTTAEILVTGIKVVDLLAPYARGGKIGLFGGAGVGKTVFIQELI0NNIAKAHGGYSVF
          	****:**:************************************** *************

MG03185.1 	TGVGERTREGNDLYHEMQETSVIQLDGESKVSLVFGQMNEPPGARARVALTG2LTVAEQF
NCU05430.1	TGVGERTREGNDLYHEMQETSVIQLDGDSKVALVFGQMNEPPGARARVALTG~LTIAEYF
FG04312.1 	TGVGERTREGNDLYHEMQETSVIQLDGESKVALVFGQMNEPPGARARVALTG0LTVAEYF
AN2315.1  	TGVGERTREGNDLYHEMQETGVIQLDGESKVSLVFGQMNEPPGARARVALTG~LTIAEYF
          	********************.******:***:******************** **:** *

MG03185.1 	RN-EGQDV~LLFIDNIFR2FTQAGSEVSALLGRIPSAVGYQPTLAVDMGGMQERITTTQK
NCU05430.1	RDEEGQDV1LLFIDNIFR~FTQAGSEVSALLGRIPSAVGYQPTLAVDMGQMQERITTTTK
FG04312.1 	RDAEGQDV~LLFIDNIFR~FTQAGSEVSALLGRIPSAVGYQPTLAVDMGGMQERITTTQK
AN2315.1  	RDEEGQDV~LLFIDNIFR~FTQAGSEVSALLGRIPSAVGYQPTLAVDMGGMQERITTTTK
          	*: ***** ********* ****************************** ******** *

MG03185.1 	GSITSVQAVYVPADDLTDPAPATTFAHLDATTVLSRGISELGIYPAVDPLDSKSRMLDPR
NCU05430.1	GSITSVQAVYVPADDLTDPAPATTFAHLDATTVLSRGISELGIYPAVDPLDSKSRMLDPR
FG04312.1 	GSITSVQAVYVPADDLTDPAPATTFAHLDATTVLSRGISELGIYPAVDPLDSKSRMLDPR
AN2315.1  	GSITSVQAVYVPADDLTDPAPATTFAHLDATTVLSRGISELGIYPAVDPLDSKSRMLDPR
          	************************************************************

MG03185.1 	IVGQEHYEVATRVQQILQEYKSLQDIIAILGMDELSEADKLTVERARKIQRFLSQPFTVA
NCU05430.1	IVGQEHYETATRVQQILQEYKSLQDIIAILGMDELSEADKLTVERARKIQRFLSQPFTVA
FG04312.1 	VVGQEHYDTATRVQQILQEYKSLQDIIAILGMDELSEADKLTVERARKIQRFLSQPFTVA
AN2315.1  	IVGEEHYNTATRVQQMLQEYKSLQDIIAILGMDELSEADKLTVERARKLERFLSQPFTVA
          	:**:***:.******:********************************::**********

MG03185.1 	QVFTGIEGKLVDLKDTIASFKAILAGEGDSLPEG~AFYMVGDLASAREKGEKILAELEKD
NCU05430.1	QVFTGIEGKLVDLKDTIASFKAILAGEGDDLPEG~AFYMVGDFASARAKGEKILAELEGQ
FG04312.1 	QVFTGIEGKLVDLKETINSFKAILSGEGDNLPEG~AFYMVGDFASAKAKGEKILAELEGQ
AN2315.1  	QVFTGIEGKLVDLKDTIASFKAIMNGEGDDLPEA1AFYMVGDLASARAKGEKILADLAKN
          	**************:** *****: ****.***. *******:***: *******:*  :

MG03185.1 	A
NCU05430.1	A
FG04312.1 	-
AN2315.1  	-
          	
```
